# *De novo* Transcriptome Analysis of *Sinapis alba* in Revealing the Glucosinolate and Phytochelatin Pathways

**DOI:** 10.3389/fpls.2016.00259

**Published:** 2016-03-04

**Authors:** Xiaohui Zhang, Tongjin Liu, Mengmeng Duan, Jiangping Song, Xixiang Li

**Affiliations:** Key Laboratory of Biology and Genetic Improvement of Horticultural Crops, Ministry of Agriculture, Institute of Vegetables and Flowers, Chinese Academy of Agricultural SciencesBeijing, China

**Keywords:** transcriptome, *Sinapis alba*, glucosinolate, phytochelatin, SSR marker, deep sequencing

## Abstract

*Sinapis alba* is an important condiment crop and can also be used as a phytoremediation plant. Though it has important economic and agronomic values, sequence data, and the genetic tools are still rare in this plant. In the present study, a *de novo* transcriptome based on the transcriptions of leaves, stems, and roots was assembled for *S. alba* for the first time. The transcriptome contains 47,972 unigenes with a mean length of 1185 nt and an N50 of 1672 nt. Among these unigenes, 46,535 (97%) unigenes were annotated by at least one of the following databases: NCBI non-redundant (Nr), Swiss-Prot, Kyoto Encyclopedia of Genes and Genomes (KEGG) pathway, Gene Ontology (GO), and Clusters of Orthologous Groups of proteins (COGs). The tissue expression pattern profiles revealed that 3489, 1361, and 8482 unigenes were predominantly expressed in the leaves, stems, and roots of *S. alba*, respectively. Genes predominantly expressed in the leaf were enriched in photosynthesis- and carbon fixation-related pathways. Genes predominantly expressed in the stem were enriched in not only pathways related to sugar, ether lipid, and amino acid metabolisms but also plant hormone signal transduction and circadian rhythm pathways, while the root-dominant genes were enriched in pathways related to lignin and cellulose syntheses, involved in plant-pathogen interactions, and potentially responsible for heavy metal chelating, and detoxification. Based on this transcriptome, 14,727 simple sequence repeats (SSRs) were identified, and 12,830 pairs of primers were developed for 2522 SSR-containing unigenes. Additionally, the glucosinolate (GSL) and phytochelatin metabolic pathways, which give the characteristic flavor and the heavy metal tolerance of this plant, were intensively analyzed. The genes of aliphatic GSLs pathway were predominantly expressed in roots. The absence of aliphatic GSLs in leaf tissues was due to the shutdown of *BCAT4, MAM1*, and *CYP79F1* expressions. Glutathione was extensively converted into phytochelatin in roots, but it was actively converted to the oxidized form in leaves, indicating the different mechanisms in the two tissues. This transcriptome will not only benefit basic research and molecular breeding of *S. alba* but also be useful for the molecular-assisted transfer of beneficial traits to other crops.

## Introduction

*Sinapis alba*, known as yellow mustard or white mustard, is an important cruciferous crop widely used as food condiments in the world (Hemingway, 1995). It has many desirable agronomic traits, such as tolerance or resistance to drought, disease, pests, and pod-shattering (Thompson, 1963; Bodnaryk and Lamb, 1991; Brown et al., 1997; Lee et al., 2014), making it an attractive resource for oil crop breeding (Tian et al., 2014). The genetically close relationship and the ease of forming hybrids between *S. alba* and *Brassica* plants make it a potential donor of resistant and other agronomic traits to *Brassica napus* and other *Brassica* crops (Brown et al., 1997; Jiang et al., 2013; Lee et al., 2014). Recently, the discovery of anti-bacterial, antioxidant, and anticancer agents in the seed extract of *S. alba* increased the interest in this plant and expanded its application beyond spices (Zielniok et al., 2015).

The spicy “heat” sensation of the *S. alba* seed powder is caused by the hydrolysis products of glucosinolates (GSLs; Hemingway, 1995; Javidfar and Cheng, 2013). The anti-bacterial and carcinogenesis-inhibiting activities of this plant are also attributed to the GSLs and their derivatives (Peng et al., 2014). Some GSL-hydrolyzed products, such as 4-methylsulfanyl-3-butenyl isothiocyanate, have been experimentally proven of their potential chemo- and cancer-prevention abilities (Abdull Razis et al., 2012). These applications exploit the advantages of GSLs. However, a high GSL content is a defective trait when attempting to use *S. alba* as an oil seed crop. Thus, GSL is a primary trait for this plant, and modulating the GSL type and its content for different application goals is important for breeding processes. GSLs belong to a type of nitrogen- and sulfur-containing plant secondary metabolite that widely exist in the order Brassicales (Fahey et al., 2001; Grubb and Abel, 2006). The GSL metabolic pathway has been extensively investigated in *Arabidopsis* and has been well studied in *B. rapa*, broccoli, radish, etc. by genome wide homologous analysis (Wittstock and Halkier, 2002; Zang et al., 2009; Wang et al., 2011; Liu et al., 2014; Pino Del Carpio et al., 2014; Wiesner et al., 2014; Mitsui et al., 2015). In *S. alba*, many GSLs have been identified (Agerbirk et al., 2008; Popova and Morra, 2014; Vastenhout et al., 2014), and many studies on the functions of GSLs have been reported (Abdull Razis et al., 2012; Peng et al., 2014). Furthermore, a QTL mapping of GSL contents has been carried out (Javidfar and Cheng, 2013). However, knowledge on the metabolic pathway of this plant is still limited.

In addition to the applications mentioned above, *S. alba* can also be used as a phytoremediation plant due to its outstanding ability to absorb cadmium (Cd) and its high biomass productivity (Plociniczak et al., 2013). Cd tolerance is potentially related to a series of biological characteristics and physiological processes, such as barriers by cell walls or mycorrhizas, reduced uptake, or efflux pumping by plasma membranes, chelation by phytochelatins, or metallothioneins, and compartmentation to vacuoles (Hall, 2002). Phytochelatin is believed to be one of the most important factors mediating Cd tolerance by chelating the heavy metal and facilitating its transport to storage locations in order to avoid cell toxicity (Cobbett, 2000; Mendoza-Cázatl et al., 2011). Phytochelatin is a stretch of (γ-Glu-Cys)_n_-Gly peptides produced in plants not via translation but by a biosynthesis process catalyzed by γ-glutamylcysteine dipeptidyl transpeptidase (phytochelatin synthase), with glutathione as the substrate (Grill et al., 1989; Cobbett, 2000).

Despite, its agricultural importance and prospective applications, genetic research on *S. alba* is far behind that of other cruciferous crops such as *B. napus* and *B. rapa*, and publicly released sequence data are rare. Though, Illumina sequencing technology has been rapidly developing and has been successfully used for many years, to the best of our knowledge, only one RNA-Seq project on *S. alba* has been published. That study profiled the differential expressions of *S. alba* leaves between drought and water recovery conditions using a transcript-end sequencing strategy in which *de novo* transcriptome assembly was not applicable (Dong et al., 2012). In the present study, a *de novo* transcriptome assembly was carried out by Illumina sequencing of mRNAs from root, stem, and leaf tissues. The transcriptome was annotated, and SSRs were identified to facilitate the application. Additionally, the pathways of GSLs and phytochelatins were analyzed.

## Materials and methods

### Plant materials and RNA extraction

**Figure 1:**
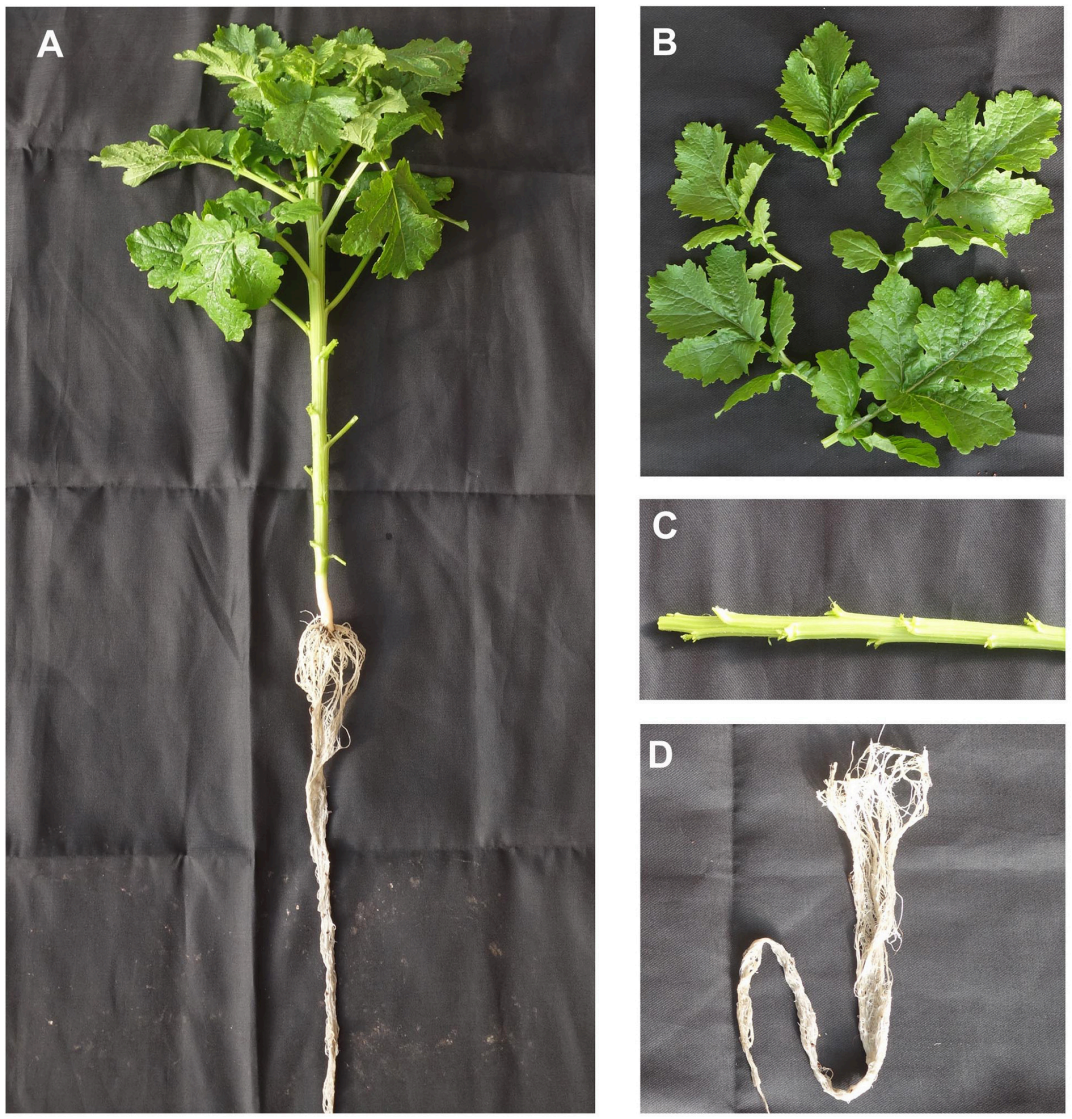
**Plants and sampling. (A)** Whole plant for sampling. **(B)** Leaf sample. **(C)** Stem sample. **(D)** Root sample.

For transcriptome sequencing, *S. alba* (ZYZ-1553) was sown in plastic pots (20 cm wide × 20 cm deep) filled with a mixture of peat soil (peat:moss:perlite:vermiculite soil = 3:2:1:1). Each pot contained one plant and was placed in a plastic tunnel located at the experimental farm of the Institute of Vegetables and Flowers, Chinese Academy of Agricultural Sciences, Beijing, China. Plants were regularly watered and fertilized. The sowing date was September 25th, and the sampling date was November 10th 2013. The leaf, stem, and root tissues were sampled from three individual plants at vegetative developmental stage (Figure 1) and then snap-frozen in liquid nitrogen and kept at −80°C for further use. Total RNA was extracted using the TRIzol reagent (Invitrogen, USA). DNase (Promega, USA) was used to remove potential DNA contamination.

For Quantitative PCR (qPCR) analysis to backup the transcriptome profiling, the plants were grown in a greenhouse with similar condition in the winter of 2015. Total RNAs were isolated from three independent plants with the same method.

### cDNA library construction and illumina sequencing

Total RNA (10 μg) was subjected to poly-A selection, fragmentation, random priming, and first and second strand cDNA synthesis with the Illumina Gene Expression Sample Prep kit (CA, USA). The cDNA fragments were subjected to an end repair process and then ligated to adapters. The products were enriched with PCR, and the fragments harboring 330-bp inserts were purified with 6% TBE PAGE gel electrophoresis. After denaturation, the single-chain fragments were fixed onto the Solexa Sequencing Chip (Flowcell) and consequently grown into single-molecule cluster sequencing templates through in situ amplification on the Illumina Cluster Station. Double-end pyrosequencing was performed on the Illumina Genome Analyzer platform with read lengths of 100 bp for each end.

### Assembly

Raw reads were first subjected to purification by removal of adaptors and low quality reads. The clean reads of leaf, stem, and root tissues were separately subjected to transcriptome *de novo* assembly using the short-read assembling program Trinity (Grabherr et al., 2011). The longest assembled sequences were termed as contigs. The paired-end reads were then mapped back to the contigs. Sequences without gaps and could not be extended at either end were defined as transcripts. The transcripts were then assembled into unigenes by filtering out redundant sequences and further assembled using TGI Clustering Tool (TGICL; Pertea et al., 2003). The unigenes from the three samples were clustered again; the longest sequences from the three data sets were adopted to form a single set of non-redundant unigenes. The unigenes were divided into two classes: Clusters including several unigenes with more than 70% similarity were prefixed with CL and suffixed with an ID number, and singletons that could not cluster to other genes were prefixed Unigene and followed with an ID number suffix.

### Annotation

We searched all unigene sequences against protein databases (Nr, Swiss-Prot, KEGG, and COG) using BLASTX (e- < 10^−5^). Protein function information was predicted from annotation of the most similar proteins in those databases. Proteins with the highest ranks in the BLAST results were obtained to determine the coding region sequences (CDS) of the unigenes, after which CDS were translated into amino sequences using the standard codon table. Unigenes that could not be aligned to any database were scanned by ESTScan (Iseli et al., 1999), producing the nucleotide sequence (5′–3′) direction and amino sequence of the predicted coding region.

### Expression levels

Unigene expression levels were calculated using the reads per kilobase per million (RPKM) method (Mortazavi et al., 2008), and the formula used is RPKM = (1,000,000 ^*^ C) / (N ^*^ L ^*^ 1000), where RPKM(A) is the expression of gene A, C is the number of reads that uniquely align to gene A, N is the total number of reads that uniquely align to all genes, and L is the length of gene A. Statistical comparisons between two samples were performed using the IDEG6 software (Romualdi et al., 2003). The general Chi squared method was used, and the false discovery rate (FDR) was applied to determine the threshold of the *Q*-value. Unigenes were considered differentially expressed (DEG) when the RPKM between two samples displayed a more than two-fold change, with an FDR < 10^−3^.

### GO and KEGG enrichments

The differentially expressed genes (DEGs) were mapped to GO terms and KEGG pathways and then subjected to an enrichment analysis using a hypergeometric test to find over-represented GO terms and KEGG pathways. The algorithm used is described as follows:

$$\begin{array}{l} P=1-\overset{m-1}{\underset{i=0}{\sum }}\frac{\left(\begin{array}{c} M\\ i \end{array}\right)\left(\begin{array}{c} N-M\\ n-i \end{array}\right)}{\left(\begin{array}{c} N\\ n \end{array}\right)} \end{array}$$

where N is the number of all genes with GO or KEGG annotation, n is the number of DEGs in N, M is the number of all genes that are annotated to certain GO terms or KEGG pathways, and m is the number of DEGs in M. The calculated *p*-value goes through Bonferroni Correction (Abdi, 2007), taking a corrected *p* ≤ 0.05 as the threshold.

### Simple sequence repeat (SSR) mining

The MIcroSAtellite identification tool MISA (http://pgrc.ipk-gatersleben.de/misa/) was used to identify and localize SSRs in unigenes longer than 1 kb. The SSR-containing sequences were extracted with a 300-bp (if < 300 bp, extracted from the end) fragment upstream and downstream of the SSR region sequence to facilitate primer design. SSR primers were design by Primer 3.0 (Untergasser et al., 2012).

### Identification of glucosinolate and phytochelatin pathways

For the glucosinolate pathway, candidate genes were first identified by BLASTN (*e* < 10^−100^) search of *S. alba* unigenes using *Arabidopsis* glucosinolate biosynthesis and transcription factors as baits. Sequences representing the complete set of glucosinolate biosynthetic and regulator genes in *A. thaliana* were acquired from the TAIR database (www.arabidopsis.org). Unigenes annotated (*e* < 10^−10^) to the KEGG glucosinolate biosynthesis reference pathway (map00966) were identified. The candidate genes were finally identified from these two selections by a manual check.

For glutathione pathways, the genes were identified from KEGG annotation to the glutathione metabolism reference pathway (map00480) with *e* < 10^−100^. Only the two directly related cycling pathways were adopted in this study. The phytochelatin synthase was identified from KO (K05941) annotation (*e* = 0).

### Quantitative PCR (qPCR)

Total RNAs (800 ng) were synthesized to first-strand cDNAs templates using *EasyScript* One-Step gDNA Removal and cDNA Synthesis SuperMix (TransGen, Beijing, China). Experiments were performed on a *Mastercycler ep realplex* Real-Time PCR System (Eppendorf, Germany) using *TransStar* Green qPCR SuperMix (TransGen). The genes and primers were listed in Table S1. The reaction volume was 25 μL, including 0.5 μL of 10 mM Forward and Reverse primer, respectively, 12.5 μL of 2 × *TransStart* Green qPCR SuperMix, 2.0 μL of the cDNA templates, 0.5 μL of Passive Reference Dye I, and 9 μL of ddH_2_O. The thermal cycling profile was: 95°C for 30 s; 40 cycles of 95°C for 10 s, 58°C for 15 s, 72°C for 10 s; then 95°C for 15 s, 60°C for 1 min, ramping to 95°C for 15 s. Three independent biological and two technical replicates were performed. *GAPDH* was used as an internal control. The relative expression levels were estimated by the 2^−ΔΔCT^ method.

## Results and discussion

### Transcriptome sequencing and assembly

**Figure 2:**
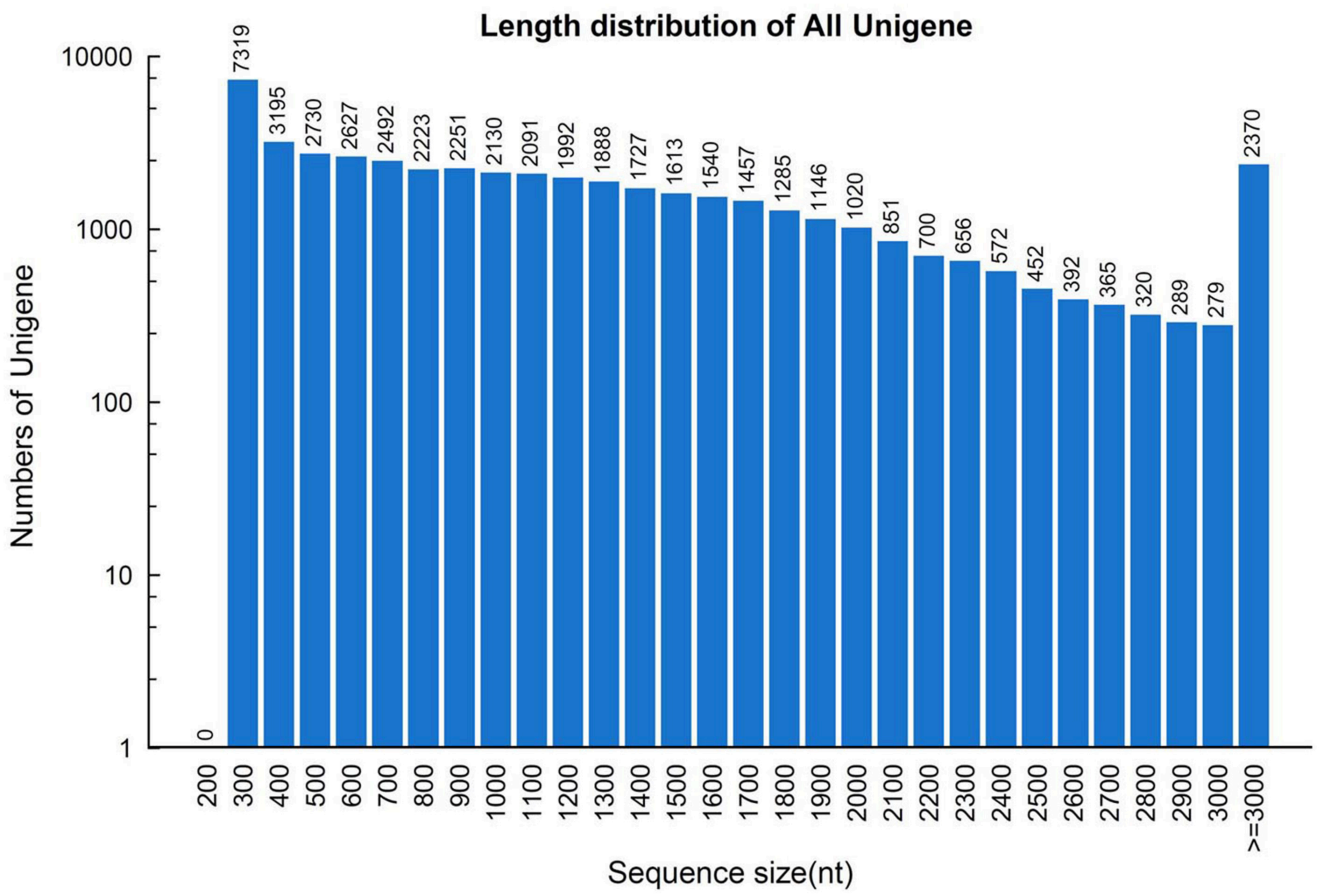
**Length distribution of the unigene assembly in the *Sinapis alba* transcriptome**.

To construct a *de novo* transcriptome database, three mRNA libraries were generated from the root, stem and leaf tissues of *S. alba* by Illumina sequencing. ~26.3, 27.9, and 26.4 million paired-end reads (100 bp read length) containing 5.26, 5.59, and 5.28 gigabase pairs of nucleotides were generated for the three samples, respectively, (Table 1). After filtering out low quality reads, ~24.2 million clean paired-end reads containing ~4.8 gigabase of clean nucleotides were obtained for each tissue. The overall GC percentages were 46.6–48.6% in these tissues. The reads of the three tissues were first assembled separately into three distinct sets of contigs and unigenes, which were consequently combined and further assembled into a set of 47,972 non-redundant unigenes, with a mean length of 1185 nt and a N50 length of 1672 nt (Table 1). The length distributions of the unigenes are shown in Figure 2, indicating a high quality reference transcriptome for use in future studies. All of the unigene sequences are provided in Supplementary File 1.

**Table 1:** **Summary of the assembly and annotation of the *Sinapis alba* transcriptome**.

	**Root**	**Stem**	**Leaf**
Total reads	26,300,732	27,933,350	26,397,896
Nucleotides (nt)	5,260,146,400	5,586,670,000	5,279,579,200
Clean reads	24,210,304	24,177,099	24,210,304
Clean nucleotides (nt)	4,842,060,800	4,835,419,800	4,842,060,800
GC percentage (%)	47.85	46.63	48.64
Unigene number	53,306	49,153	45,745
Mean length of unigene (nt)	900	862	760
Combined non-redundant unigene	47,972		
Total length (nt)	56,824,691		
Mean length (nt)	1185		
N50 (nt)	1672		
Nr	44,485 (92.73%)		
Nt	46,140 (96.18%)		
GO	41,796 (87.13%)		
COG	18,906 (39.41%)		
Swiss-Prot	31,614 (65.9%)		
KEGG	27,323 (56.96%)		
All annotated	46,535 (97%)		

### Functional annotation

**Figure 3:**
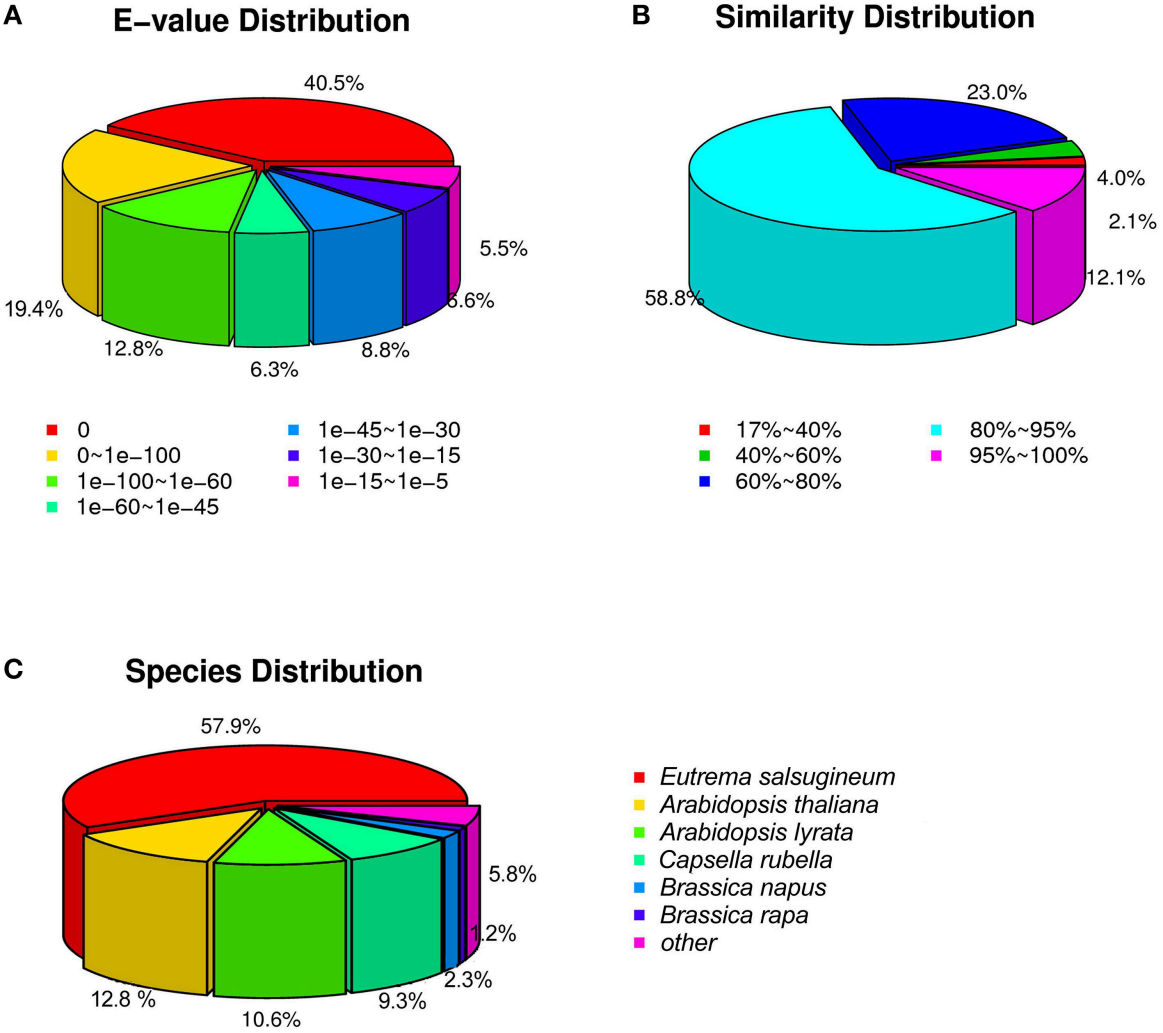
**The distribution of unigenes annotated to the NCBI non-redundant nucleotide sequence (Nt) database. (A)** *E*-value distribution. **(B)** Similarity distribution. **(C)** Species distribution.

We screened the unigene sequences against the NCBI non-redundant (Nr), Swiss-Prot, Kyoto Encyclopedia of Genes and Genomes (KEGG) pathway, Gene Ontology (GO), and Clusters of Orthologous Groups of proteins (COGs) protein databases using BLASTX (*e* < 10^−5^). Unigenes were also searched against the NCBI non-redundantnucleotide sequence (Nt) database using BLASTN (*e* < 10^−5^). Protein function was predicted from the annotations of the most similar proteins in those databases. In total, 46,535 (97.0%) of the 47,972 unigenes were annotated by at least one of these databases (Table 1). Amongst, 44,485 (92.7%) unigenes were annotated by Nr. As shown in Figure 3, more than 87.8% of the unigenes were annotated with an *e* < 10^−30^, and more than 70.9% of the unigenes contains more than 80% similarity to the reference genes in the database, indicating that the annotations are reliable. ~94.2% of the unigenes were annotated to cruciferous plants (Figure 3C).

**Figure 4:**
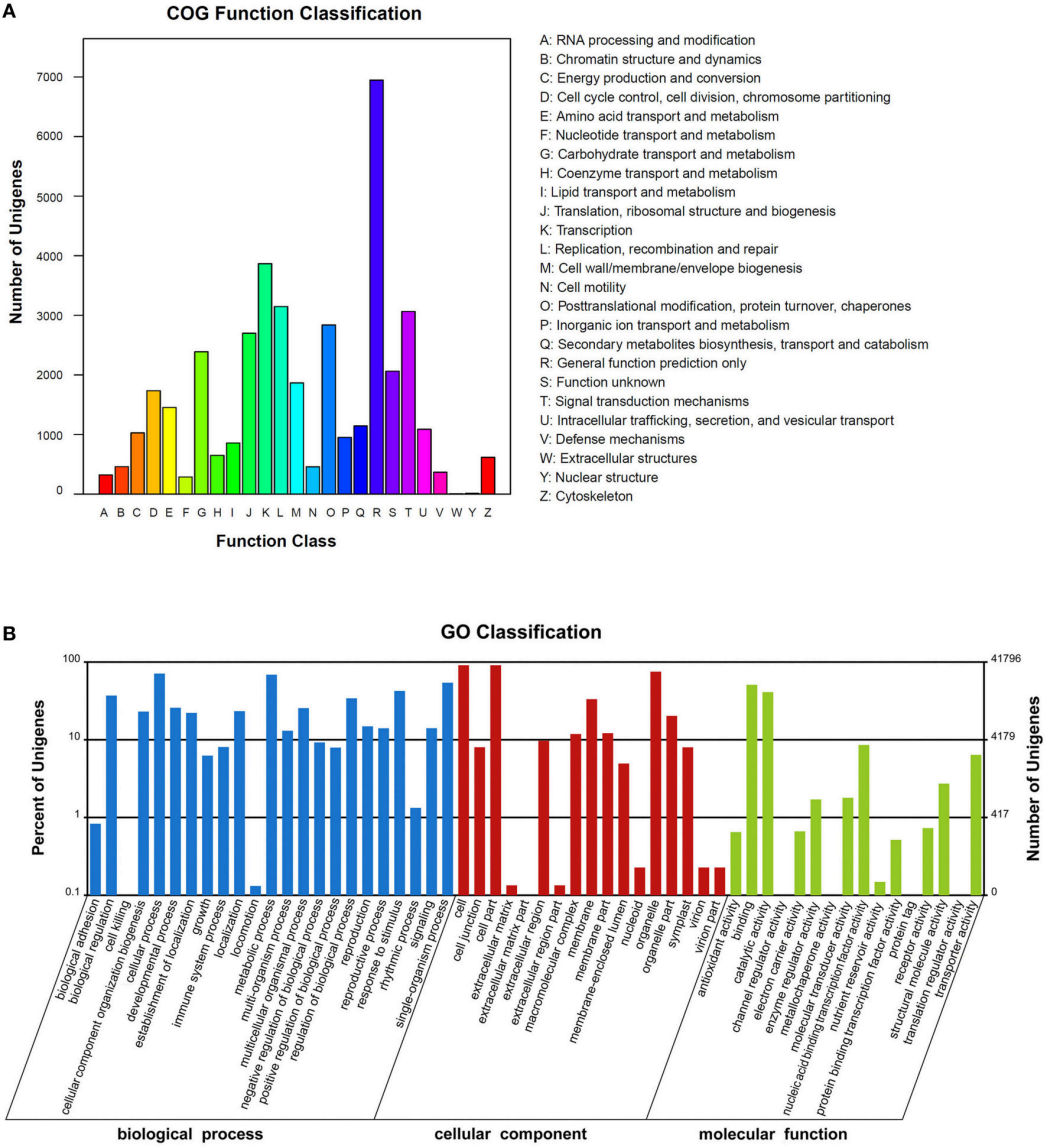
**The clusters of unigenes of the *Sinapis alba* transcriptome. (A)** Clusters of Orthologous Groups of proteins (COGs) annotation. **(B)** Gene Ontology (GO) terms.

COGs annotation indicated that 18,906 (39.4%) unigenes were assigned to one or more COG functional classes. The most abundant class was “general function prediction only,” including 6946 (36.7% of the annotated COGs) unigenes, followed by the classes “transcription” (3866; 20.5%) and “replication, recombination and repair” (3146; 16.6%; Figure 4A). Functions of 41,796 (87.1%) unigenes were further classified by Gene Ontology (GO) analysis. The largest GO terms found in the “biological process” ontology were “cellular process” and “metabolic process,” comprising 71.1 and 68.7% of the GO-termed unigenes, respectively. In the “cellular component” and “molecular function” ontologies, the top terms were “cell (or cell part)” and “binding,” which are 90.9 and 50.9% of the total unigenes annotated by GO, respectively, (Figure 4B). KEGG metabolic pathway analysis revealed that 27,323 (56.96%) unigenes could be assigned to 128 pathways (level 3). The most abundant pathways are metabolic, secondary metabolite biosynthesis, and plant hormone signal transduction, comprising 5891 (21.56%), 2868 (10.5%), and 1853 (6.78%) unigenes, respectively, (Supplementary File 2). All of the above annotations to each unigene are integrated in Table S2.

The orientation and coding sequence (CDS) of 43,953 unigenes were determined by BLASTX (*e* < 10^−5^) to Nr, Swiss-Prot, KEGG, and COG databases. Those unigenes that had no blast hit to any database were analyzed by ESTScan, in which 502 additional unigenes were assigned an orientation and a CDS. The encoding proteins were deduced from the CDSs using the standard codons, and the protein sequences are shown in Supplementary File 3.

### Tissue patterns

**Figure 5:**
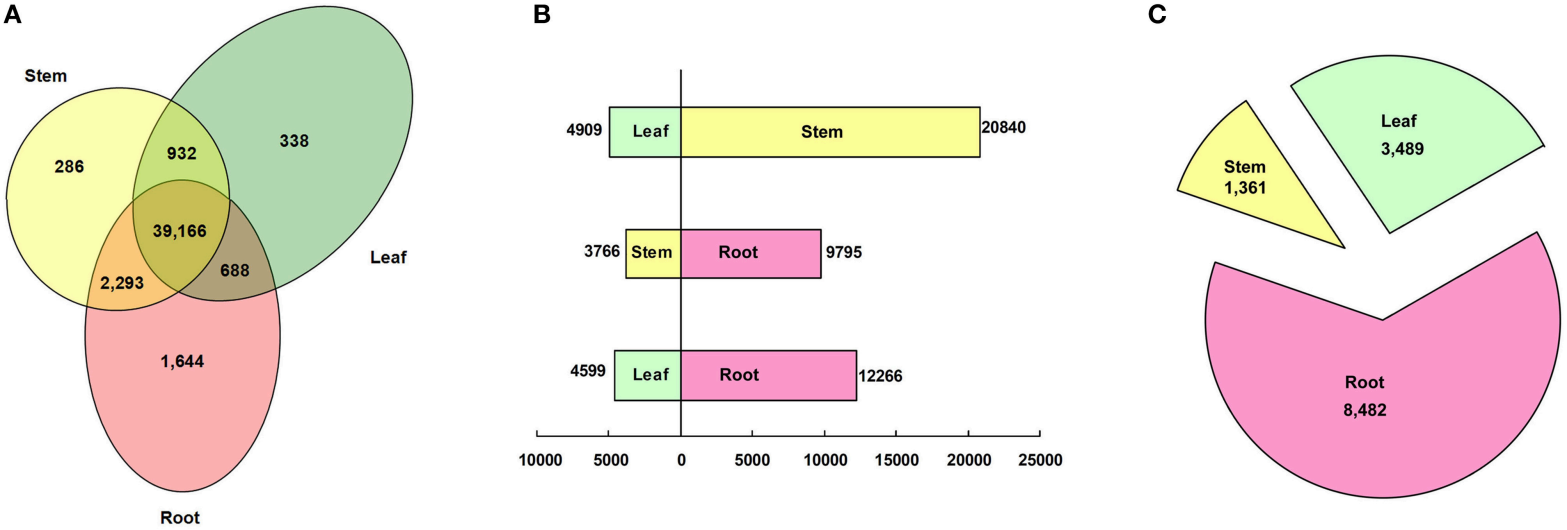
**Classification of tissue patterns. (A)** Venn diagram of tissue-specific gene expression. **(B)** Histogram of differentially expressed genes between tissues. **(C)** Pie chart of genes predominantly expressed in each tissue.

To profile the expressional tissue patterns, we first aligned the reads back to the unigenes; the reads aligned to mono sites were counted for expression calculations. 1644, 286, and 338 unigenes were specifically expressed in roots, stems and leaves, respectively, (Figure 5A). The fact that more genes were specifically expressed in roots than in stems and leaves indicated, that the root system faced more complications and performed many specific functions due to rhizosphere microbe. The root-specific genes included a large fraction of transcription factors (72, 4.38%) and genes related to phytohormone (33, 2.01%) and materials transport (102, 6.20%; Table S3). The transcription factors were composed of 33 MYBs, 22 MADS-boxes (including 10 AP2 and 7 EREBP-like), 7 WRKYs, 4 TGA, 3 homeobox-leucine zipper proteins and 2 MYC2. The root-specific phytohormone genes were related to the metabolism and signal transduction of gibberellins (GA), jasmonate (JA), abscisic acid (ABA), brassinosteroid (BR), and cytokinin. The majority of root-specific transporters were comprised of 55 major facilitator super family (MFS) transporters which facilitate the transport of glucose, sugar, amino acid, peptide, histidine, zinc, nitrate/nitrite, inorganic phosphate, and organic cation, as well as serving as sodium/hydrogen exchangers and iron-regulated transporters. Other transporters included cation transport ATPase, multidrug resistance protein, ABC-type multidrug transport system, Ca^2+^/H^+^ antiporter, K^+^ transporter, magnesium transporter, ammonium transporter, amino acid transporter, copper chaperone, and vacuolar protein sorting-associated protein.

Using a threshold of RPKM > 2 and FDR < 0.001, 25,749 (53.68%), 13,561 (28.27%), and 16,865 (35.16%) unigenes were differentially expressed between the leaf and the stem, the stem and the root, and the leaf and the root, respectively, (Figure 5B). By using a hypergeometric distribution analysis, the root-stem DEGs were enriched in GO terms related to the vacuole, the cell wall, and the ER body. The stem-leaf DEGs were enriched in terms related to the plastid, chloroplast, and Golgi apparatus. The GO terms of the photosystem, plastid part, apoplast, and those integral to the membrane were significant differentially expressed in the root, stem, and leaf (Table S4). Via pathway enrichment, the inter-tissue DEGs were enriched in 50 pathways (Table S5). Aside from the “endocytosis,” “regulation of autophagy,” “SNARE interactions in vesicular transport,” “ABC transporters,” “plant-pathogen interaction,” “Circadian rhythm—plant,” and “plant hormone signal transduction” enriched in the three comparisons. All of the other 43 pathways belonged to the metabolism pathway, including “energy metabolism,” “carbohydrate metabolism,” “lipid metabolism,” “glycan biosynthesis and metabolism,” “metabolism of terpenoids and polyketides,” and “biosynthesis of other secondary metabolites.” The global maps of “metabolic pathways” and “biosynthesis of secondary metabolites,” “ether lipid metabolism,” “porphyrin and chlorophyll metabolism,” some pathways of “energy metabolism,” and “carbohydrate metabolism” were enriched in any two of the three tissue comparisons. The biosynthesis of secondary metabolites, such as phenylalanine, tryptophan, stilbenoid, diarylheptanoid and gingerol, flavones, flavonoid, indole alkaloid, and phenylpropanoid were enriched in the root-stem and the root-leaf comparisons. The biosynthesis of glucosinolate, isoflavonoid, isoquinoline and benzoxazinoid, cyanoamino acid, glutathione, and seven types of terpenoids and polyketides were enriched in the root-stem comparison. The “amino sugar and nucleotide sugar metabolism,” “ascorbate and aldarate metabolism,” “fructose and mannose metabolism,” and “pentose phosphate pathway” pathways were significantly enriched in the stem-leaf comparison.

**Figure 6:**
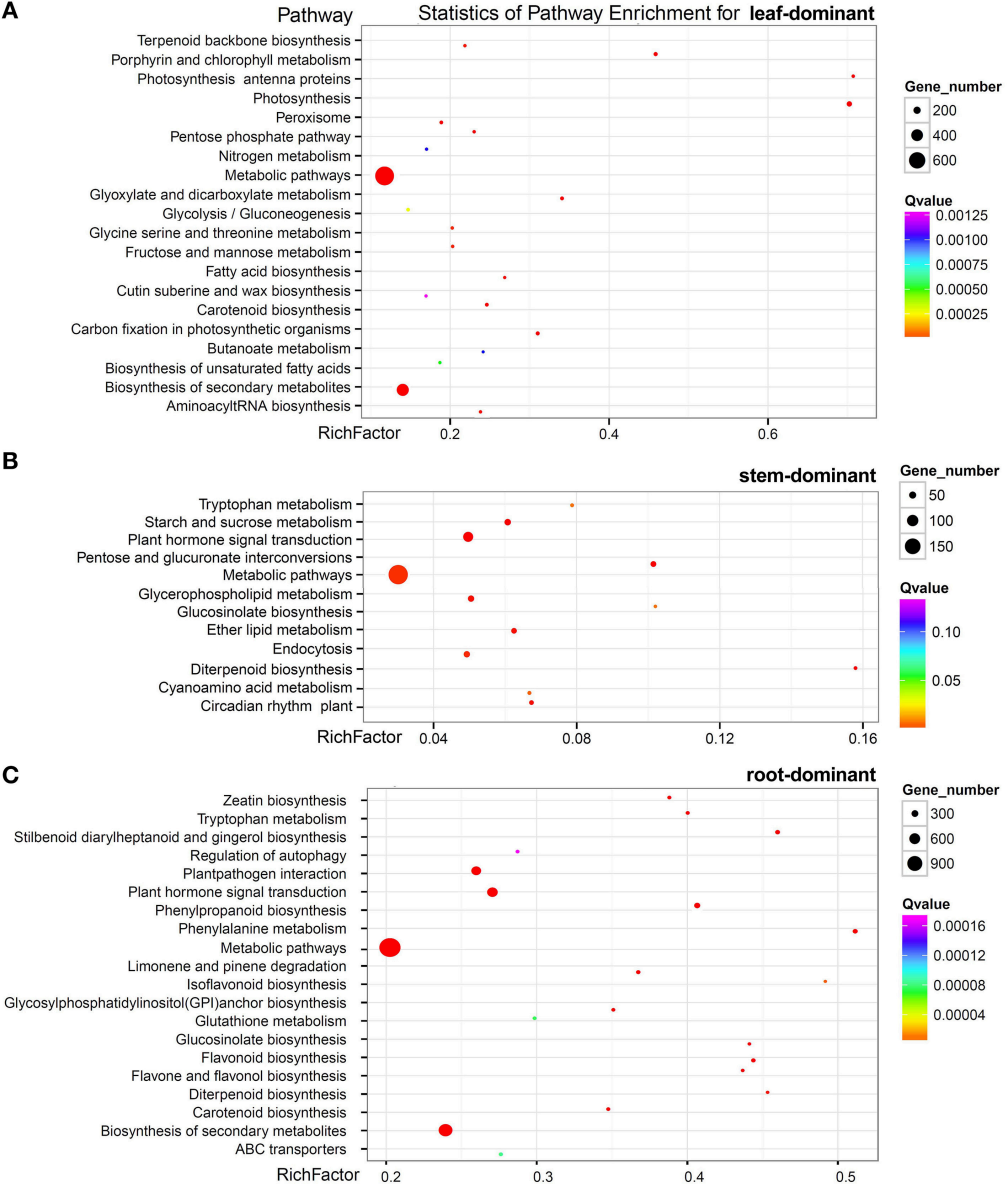
**Pathway enrichment of genes predominantly expressed in the leaf (A) the stem (B) and the root (C)**.

Further, analysis identified that 3489, 1361, and 8482 unigenes were predominantly expressed (more than a two-fold up-expression than the other two samples, FDR < 0.001) in the leaf, stem, and root, respectively, (Figure 5C). Via a hypergeometric test, the leaf-dominant genes were enriched in pathways related to photosynthesis and carbon fixation (Figure 6A). This finding is consistent with the fact that the leaf is the main photosynthesis organ. Genes predominantly expressed in the stem were not only enriched in pathways related to sugar, ether lipid, and amino acid metabolism but also in plant hormone signal transduction and circadian rhythm pathways (Figure 6B). The root dominant genes enriched in flavonoid, phenylpropanoid, and terpenoid biosynthesis pathways, plant hormone signal transduction, plant-pathogen interaction, regulation of autophagy and ABC transporters and glutathione metabolism (Figure 6C).The enrichment of the flavonoid and phenylpropanoid biosynthesis pathways indicates, that roots were actively synthesizing lignin and cellulose for rapid cell growth. The enrichment of plant-pathogen interaction pathways indicates that roots faced soil borne diseases. The enrichment of glutathione metabolism genes is an interesting phenomenon because glutathione-generated phytochelatin plays an important role in Cd absorption (Grill et al., 1989; Cobbett, 2000). The enrichment of autophagy and ABC transporters is consistent with mineral nutrient uptake, which is the primary function of roots. The ABC transporters are also responsible for transporting phytochelatin-chelated heavy metals to the vacuolar for storage and detoxification (Mendoza-Cázatl et al., 2011).

### Simple sequence repeat (SSR)

**Figure 7:**
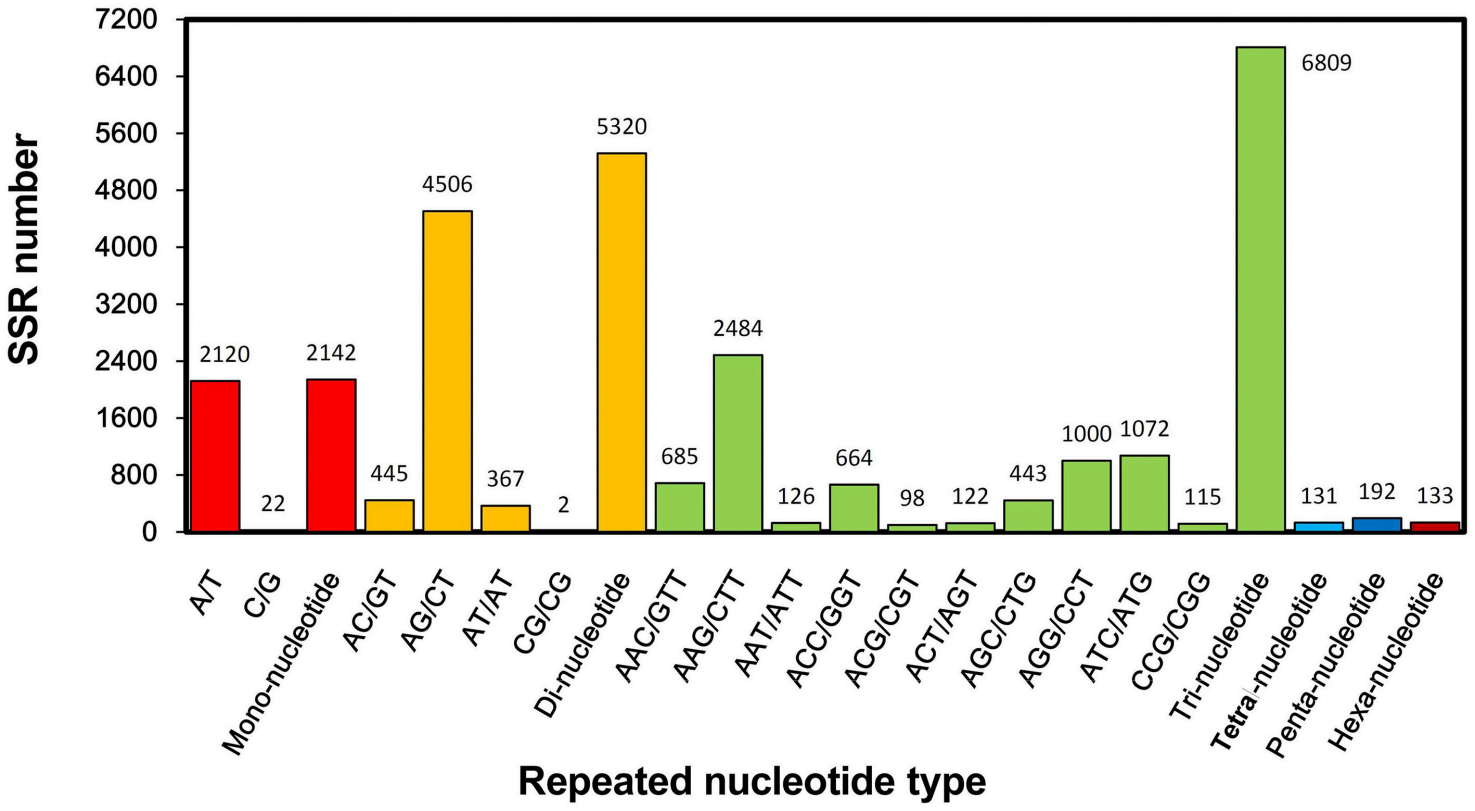
**Histogram of the statistics of simple sequence repeat (SSR) types**.

Molecular marker is important genetic tool but is still considered undeveloped for *S. alba*. Though many intron length polymorphism markers have been developed for this plant (Javidfar and Cheng, 2013), SSR is still a useful molecular marker, especially for this less-studied crop. In this study, a total of 14,727 SSRs was identified from 56,824,691 nt transcriptome sequences; nearly every 4953 nt contained one SSR. Among them, 11,473 (23.9%) of the 47,972 total unigenes contained at least one SSR, of which 2539 unigenes contained more than one SSR. The tri-, di-, and mono-nucleotide repeats were the main types, with values of 6809 (59.3%), 5320 (46.4%), and 2142 (18.7%) SSRs, respectively, (Table 2). The A/T, AG/CT, and AAG/CTT were the dominant types of the mono-, di-, and tri-nucleotide repeats (Figure 7).

**Table 2:** **Statistics of simple sequence repeat types**.

**Number of repeats**	**Mono-nucleotide repeats**	**Di-nucleotide repeats**	**Tri-nucleotide repeats**	**Tetra-nucleotide repeat**	**Penta-nucleotide repeats**	**Hexa-nucleotide repeats**
4	0	0	0	0	175	133
5	0	0	4299	104	17	0
6	0	2104	1755	27	0	0
7	0	1302	682	0	0	0
8	0	891	71	0	0	0
9	0	530	1	0	0	0
10	0	335	1	0	0	0
11	0	145	0	0	0	0
12	669	13	0	0	0	0
13	467	0	0	0	0	0
14	336	0	0	0	0	0
15	188	0	0	0	0	0
16	152	0	0	0	0	0
17	93	0	0	0	0	0
18	55	0	0	0	0	0
19	44	0	0	0	0	0
20	13	0	0	0	0	0
21	37	0	0	0	0	0
22	45	0	0	0	0	0
23	36	0	0	0	0	0
24	7	0	0	0	0	0
Subtotal	2142	5320	6809	131	192	133

To facilitate applications, 12,830 pairs of primers were developed for 2522 SSR-containing unigenes (Table S6). Twenty (0.16%), 2579 (20.1%), 9873 (77%), 84 (0.65%), 119 (0.93%), and 115 (1.21%) pairs of primers were designed for detecting fragments harboring mono-, di-, tri-, tetra-, penta-, and hexa- nucleotide repeats. To the best of our knowledge, this is the first large collection of SSR markers for this plant.

### Glucosinolate metabolic pathway in *S. alba*

**Figure 8:**
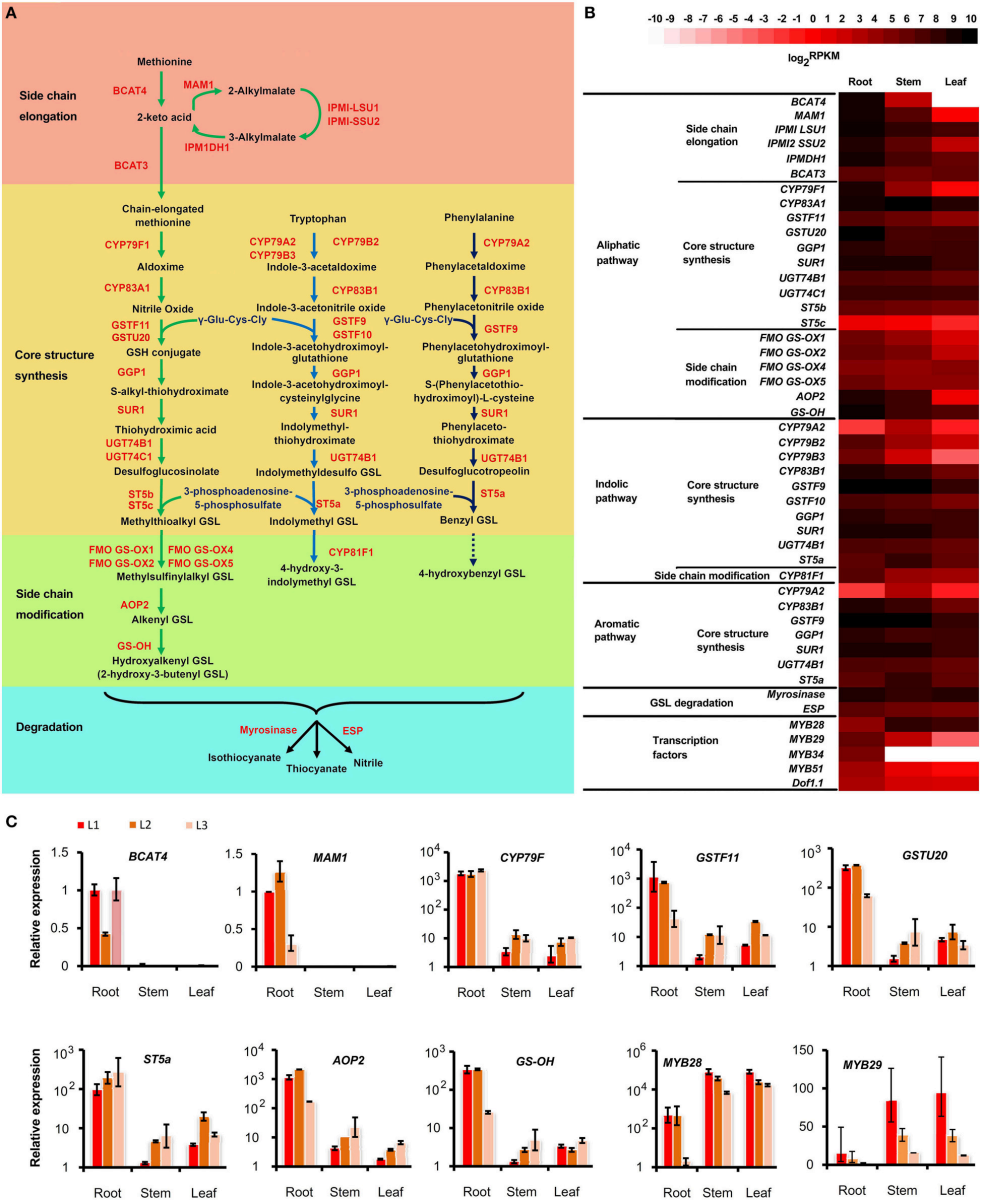
**Glucosinolate (GSL) metabolic pathway and its tissue pattern in *Sinapis alba.* (A)** Pathway map. **(B)** Heatmap showing the expression level of genes encoding the enzymes and regulators in the GSL pathway. **(C)** qPCR results. Bars indicating SDs of technical replicates. BCAT, branched-chain amino acid aminotransferase; MAM, methylthioalkylmalate synthase; IPMI LSU1, Isopropylmalate Isomerase Large Subunit 1; IPMI SSU2, Isopropylmalate Isomerase Small Subunit 2; IPMDH, Isopropylmalate Dehydrogenase; CYP, Cytochromes P450; GSTF, Glutathione S-transferases; GGP, Gamma-Glutamyl Peptidase; SUR, C-S lyase SUPERROOT; UGT, UDP-glucosyltransferase; ST, Desulfoglucosinolate Sulfotransferases; FMO-GSOX, Flavin-Monooxygenase Glucosinolate S-Oxygenase; AOP, Alkenyl hydroxalkyl producing; GS-OH, Fe (II)-dependent oxygenase superfamily protein; ESP, epithiospecifier protein.

Glucosinolate (GSL) is an important metabolite that confers the special pungent properties of *S. alba* seeds (Hemingway, 1995; Javidfar and Cheng, 2013). Most of the glucosinolate in *S. alba* is 4-hydroxybenzyl, 3-indolylmethyl, 4-hydroxy-3-indolylmethyl, and 2-hydroxy-3-butenyl GSL (Javidfar and Cheng, 2013). Therefore, all three GSL metabolic pathways, i.e., the aliphatic, indolic, and aromatic GSL pathways, must exist in *S. alba*. To investigate the molecular basis for the GSL biosynthesis in this plant, the transcripts of the GSL pathways were identified in the *S. alba* transcriptome by KEGG annotation and by BLAST search for homologous genes of the *Arabidopsis* GSL pathway. A total of 71 transcripts were identified as candidate genes for 32 enzymes of GSL biosynthesis and degradation pathways (Table S7). A deduced GSL metabolic pathway map was constructed for *S. alba* (Figure 8A). The pathway is comprised of four stages including side chain elongation, core structure synthesis, side chain modification, and degradation of GSLs. The side chain elongation process was investigated thoroughly only for methionine in the aliphatic GSL pathway. The methionine was firstly deaminated to form 2-keto acid by a branched-chain amino acid aminotransferase (BCAT4) and then entered two cycles of three successive transformations: (1) condensation with acetyl-CoA by methylthioalkylmalate synthase 1(MAM1), (2) isomerization by isopropylmalate isomerase large subunit 1 (IPMI LSU1) and isopropylmalate isomerase small subunit 2 (IPMI SSU2), and (3) oxidative decarboxylation by isopropylmalate dehydrogenase 1 (IPMDH1; Sønderby et al., 2010; Wang et al., 2011). After a transamination reaction catalyzed by BCAT3, two carbons were added to the side chain of methionine. Five of these six enzymes (except BCAT3) displayed significantly up-regulated expression in the root compared to the stem and the leaf (Figure 8B, Table S7). In particular, *BCAT4* was not expressed and *MAM1* was barely detectable in the leaf, indicating that the side chain elongation pathway is blocked in this organ. The side-chain elongated methionine was then subjected to core structure synthesis. Twenty-two genes that encoded 10 enzymes catalyzing the seven steps in this process were expressed (Figure 8B, Table S7). First, two cytochrome P450s (CYP79F1 and CYP83A1) successively converted the side-chain elongated methionine to nitrile oxide. Then, the molecules were conjugated to a glutathione (GSH) by two glutathione S-transferases (GSTF11 and GSTU20). *CYP79F1, GSTF11*, and *GSTU20* were highly expressed in the root, moderately expressed in the stem and only trace amounts were expressed in the leaf. This result, again, implies that the aliphatic GSLs are predominantly produced in the root but minimally synthesized in the stem and leaf of *S. alba*. This finding is consistent with a previous report that aliphatic GSLs were detected significantly in the root but minimally detected (if any) in the leaf (Agerbirk et al., 2008). GSH conjugates were deglutamylated by gamma-glutamyl peptidase 1 (GGP1) to form S-alkyl-thiohydroximate and then catalyzed by C-S lyase SUPERROOT1 (SUR1) to form thiohydroximic acids and consequently S-glucosylated by UDP-glucosyltransferase (UGT74B1&C1) to generate desulfoglucosinolates, which were finally catalyzed by desulfoglucosinolate sulfotransferases (ST5b&c) to generate the core structure of glucosinolate. The genes for these four metabolic steps were expressed in the three organs with no significant difference, indicating these reactions were not rate-limiting nodes in this pathway.

For the indolic and aromatic pathways, the side-chain elongation pathway did not exist and was not detected in this plant. The core structure synthesis processes were similar to that of the aliphatic pathway, with the only difference being that the members from a different subfamily of P450 (CYP79A2, B2, B3; CYP83B1), GST (GST9&10), and ST (ST5a) were present in the indolic (Wiesner et al., 2014) and aromatic pathways (Figure 8, Table S7). Interestingly, despite the fact that *CYP79B2*&*3* and *CYP83B1* were still expressed significantly higher in the root, some genes including *CYP79A2* and *ST5a* were expressed the highest in the stem, indicating that indolic and aromatic GSLs have differential tissue profiles compared to aliphatic GSLs. In fact, the aromatic (benzyl and 4-hydroxybenzyl) GSLs are the dominant types of GSLs that make up the largest proportion of the total GSLs in *S. alba*. The level of 4-hydroxybenzyl GSL is much higher than that of benzyl GSL, and both accumulate more in the leaf than in the root (Agerbirk et al., 2008). However, the indolic GSLs (1-methoxy-indol-3ylmethyl, 4-methoxy-indol-3ylmethyl, indol-3ylmethyl, and 4-hydroxyindol-3ylmethyl) are mainly synthesized in hairy root (Kastell et al., 2013). Thus, other unknown enzymes or regulators must exist to control the two pathways separately, aside from the predicted enzymes shared by indolic and aromatic pathways, or some multi-copy genes of these predicted shared enzymes have functionally diverged but cannot be distinguished by only sequence messages.

The GSL core structures subsequently entered side-chain modification processes. The aliphatic methylthioalkyl GSLs were first oxidized to methylsulfinylalkyl GSLs by flavin-monooxygenase glucosinolate S-oxygenase (FMO-GSOX) and then conferred to alkenyl GSLs by alkenyl hydroxalkyl producing (AOP) protein; then, they were consequently decorated with a hydroxyl group by Fe (II)-dependent oxygenase super family protein (GS-OH) to form hydroxylalkenyl GSLs, which is mainly 2-hydroxyl-3buteny GSL in *S. alba* (Figure 8B). Four *FMO-GSOXs* (*FMO-GSOX1,2,4*,&*5*), one *AOP* (*AOP1*) and one *GS-OH* were expressed in *S. alba*, and except for *FMO-GSOX4*, all of these genes were significantly expressed more in the root than the stem and the leaf (Figure 8B, Table S7). For the indolic pathway, the indolymethyl GSL was catalyzed by CYP81F1 to generate 4-hydroxy-3-indolymethyl GSL. Four transcripts of *CYP81F1* were identified in the transcriptome and were expressed higher in the root (Figure 8B, Table S7). For the aromatic pathway, the enzyme catalyzing the benzyl GSL to 4-hydroxybenzyl GSL is still unknown, with GS-OH and CYP81F as possible candidates.

GSLs are stored in vacuoles and will be released and quickly degraded to form isothiocyanates and nitriles when the cells are damaged, such as during food preparation or from pest-chewing. This process is catalyzed by the endogenous plant enzyme myrosinase and can be affected by the epithiospecifier protein (ESP) and reaction environments, such as pH and temperature (Fenwick et al., 1983; Ludikhuyze et al., 2000; Burow et al., 2006; Williams et al., 2008). The endogenous myrosinase hydrolyzes GSLs to isothiocyanates, which gives functions such as anti-insects and anti-microbes in plants and serve as potential anti-tumor compounds in the human diet (Bednarek et al., 2009; Clay et al., 2009; Øverby et al., 2015; Veeranki et al., 2015). When ESP was present, the production of isothiocyanates was reduced, and the GSLs were hydrolyzed to form thiocyanates, epithionitriles, or simple nitriles, depending on the GSL structure (Lambrix et al., 2001; Burow et al., 2009). The biological function of nitriles is still unclear. However, their toxicity effects in human diets and animal feeds are confirmed. Thus, it is important to enhance the isothiocyanate content and reduce the nitrile content in cruciferous crops. In *S. alba*, seven myrosinase and three ESP encoding transcripts were identified (Table S7). Overall, the two gene families were expressed in all of the three organs with no significant tissue bias (Figure 8B). However, four myrosinase and one ESP transcripts were specifically expressed in the root, and two myrosinase and one ESP transcripts were predominantly expressed in the stem and the leaf, indicating these genes have been functionally specialized (Table S7). Due to many high GSL-content organs, such as seeds and flowers, not being analyzed, other genes that are undetected in this study could be specifically expressed in these tissues. The multi-copy and tissue specialization properties of these two enzymes offer the possibility to fine-tune the types and amounts of GSL hydrolysis products in the target tissues and desired developmental stages.

The Dof1.1 and six members of the MYB family (MYB28, 29, 34, 51, 76, and 122) transcription factors were reported to regulate the biosynthesis of GSLs (Skirycz et al., 2006; Gigolashvili et al., 2007, 2008; Hirai et al., 2007; Sønderby et al., 2010). From our *S. alba* transcriptome, three *Dof1.1*, one *MYB28*, three *MYB29*, one *MYB34*, and one *MYB51* homologs were found (Table S7). *MYB28* was expressed higher in the stem and the leaf than in the root. *MYB29* and *MYB51* were significantly up-regulated in the root, and *MYB34* was expressed only in the root (Figure 8B, Table S7). Research results in *Arabidopsis* have shown that *MYB28* and *MYB29* are key regulators of the aliphatic GSL biosynthesis (Gigolashvili et al., 2007; Hirai et al., 2007; Sønderby et al., 2010). The expression of *MYB29* was well correlated with the tissue pattern of the aliphatic GSL synthesis genes. However, the significant up-expression in the stem and the leaf indicated that MYB28 may have acquired new roles, such as regulating indolic and aromatic GSL biosynthesis in *S. alba*.

To confirm the reliability of the transcriptome profiling, a series of qPCR analyses were performed to 10 genes which showed differential expression between tissues. As shown in Figure 8C, 8 of the 10 genes displayed similar expression patters between RNA-Seq and qPCR technology, indicating the relative high quality of the transcriptome profiling. Especially, the extreme low abundant of *BACT4* and *MAM1* in stem and leaf revealed by qPCR analysis of three independent lines strengthened the estimation that the absence of these two genes turned off the biosynthesis of aliphatic GSLs in these tissues. The two conflicting genes, *ST5a* and *MYB29*, might be developmental and environmental sensitive, due to the plants used in the two experiments were grown in two different times and places. The *MYB29* is extreme unstable because its expressions were significantly differed among the three biological replicates.

### The glutathione metabolic pathway and phytochelatin synthesis

**Figure 9:**
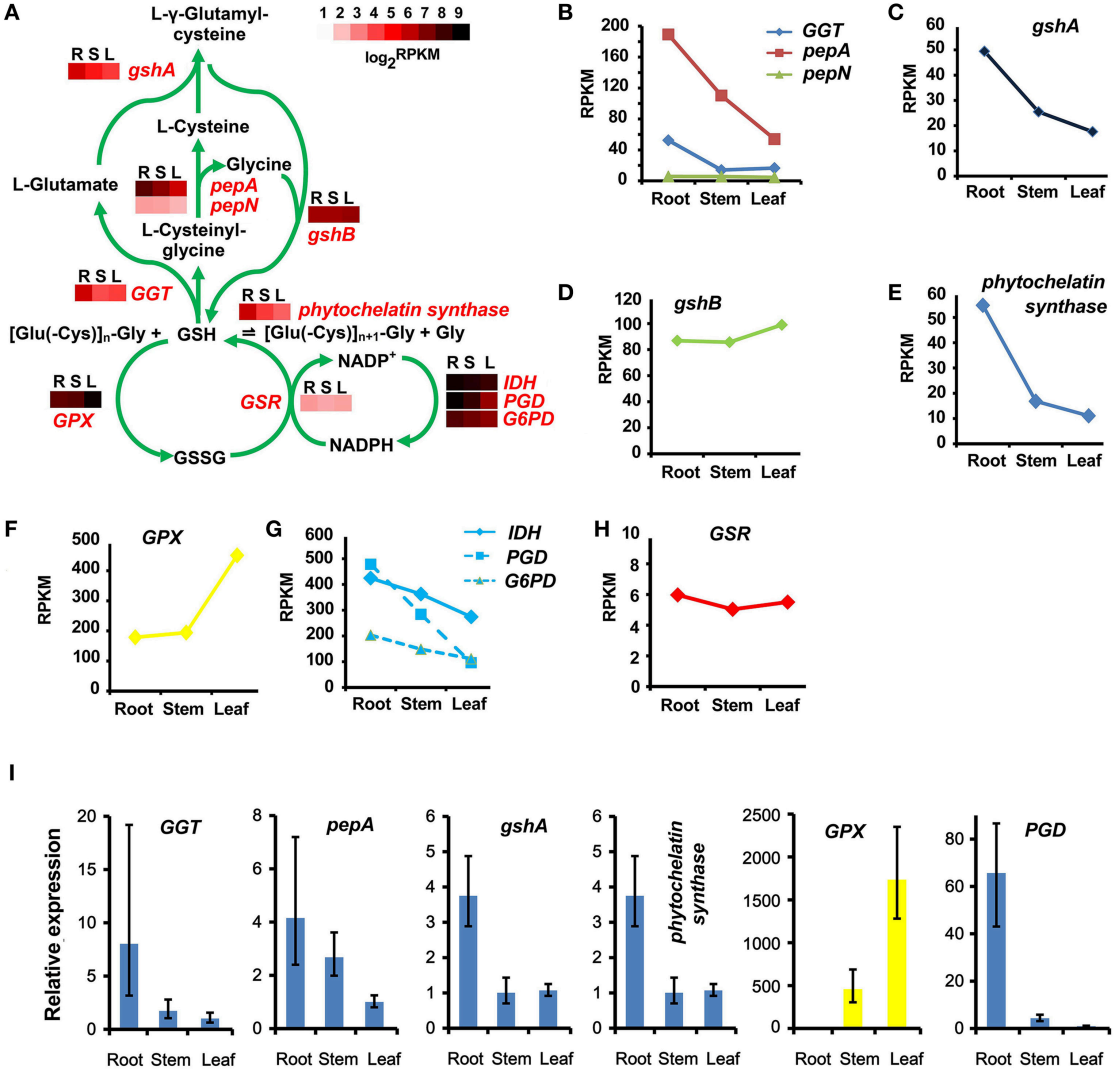
**Glutathione and phytochelatin synthetic pathways and their tissue patterns in *Sinapis alba*. (A)** The glutathione cycling and phytochelatin synthetic pathway map with heatmaps showing the expression levels of the corresponding enzymes. R, root; S, stem; L, leaf. **(B–H)** Line charts showing the gene expression levels based on RNA-seq. **(I)** qPCR results. Bars indicating SDs of three biological and two technical replicates. GGT, gamma-glutamyl transferase; pepA, leucyl aminopeptidase; pepN, aminopeptidase; gshA, γ-glutamylcysteine synthetase; gshB, glutathione synthetase; GPX, glutathione peroxidase; IDH, isocitrate dehydrogenase [NADP]; PGD, 6-phosphogluconate dehydrogenase; G6PD, glucose-6-phosphate 1-dehydrogenase; GSR, glutathione reductase.

Phytochelatin is a non-mRNA translated glutamylcysteine-repeated peptide (Grill et al., 1989). It is widely believed, that phytochelatin plays an important role in Cd tolerance in plants (Cobbett, 2000). Phytochelatin is synthesized by phytochelatin synthase, with glutathione as building blocks (Grill et al., 1989). Thus, transcripts encoding the phytochelatin synthase and in the glutathione metabolic pathway were identified in the *S. alba* transcriptome (Figure 9A). Forty-three transcripts were identified as candidate genes encoding 10 enzymes catalyzing the glutathione metabolic pathway, and two transcripts were isolated as *phytochelatin synthase* genes (Table S8). There is a synthesis-degradation cycle and an oxidation-reduction cycle in this plant (Figure 9A). The expression of *gamma-glutamyl transferase* (*GGT*) and *leucyl aminopeptidase* (*pepA*), which were responsible for the degradation of glutathione to L-glutamate, L-cysteine, and glycine, were higher in the root than in the stem and the leaf (Figures 9A,B). A similar pattern was also shown for the expression of γ*-glutamylcysteine synthetase* (*gshA*), which catalyzed L-glutamate and L-cysteine to form L-γ-glutamylcysteine (Figure 9C). However, the final glutathione biosynthesis step which was catalyzed by glutathione synthetase (gshB) with L-γ-glutamylcysteine and glycine as substrates, showed no significant differences among the three tissues in terms of expressional levels (Figure 9D). These results indicated that the glutathione synthesis-degradation cycle was elevated in the root, followed by in the stem, and it was the lowest in the leaf tissues. Degradation products, such as L-glutamate, L-cysteine and glycine, and the metabolic intermediate products, i.e., L-γ-glutamylcysteine, may be shunted to some other metabolic pathways in the root due to the upstream genes being expressed relatively higher in the root but the final step was not significantly different among the three tissues. The transcripts of *phytochelatin synthase* were three times more abundant in the root than the stem and the leaf (Figure 9E), indicating that phytochelatin was predominantly synthesized in the root. This phenomenon is in accordance with the assumption that phytochelatin played a key role in Cd detoxification, and its high accumulation in the root resulted in a high level Cd-tolerance in *S. alba*. For the oxidation-reduction cycle, the situation was completely different (Figure 9A). The genes encoding glutathione peroxidases (GPXs) were highly expressed in the leaf (Figure 9F). However, glutathione reductase (GSR) was not differentially expressed among the three organs (Figure 9H). These results indicated that in leaf tissues, glutathione was actively transformed to its oxidized form (GSSG). This transformation could occur because the leaf contains more reductive substances produced by the photosynthesis system. NADPH is a coenzyme for the reduction of GSSG to GSH. The enzymes, isocitrate dehydrogenase (IDH), 6-phosphogluconate dehydrogenase (PGD), and glucose-6-phosphate 1-dehydrogenase (G6PD), catalyzing the reduction of NADP+ to NADPH were expressed higher in the root than in the stem and were expressed the lowest in the leaf (Figure 9G), which could drive the reduction process with the corresponding speed in these organs. Finally, glutathione was extensively oxidized in leaf tissues, while reduced forms were more present in the root tissues, and were actively converted into phytochelatin. The expression patterns of the genes in this pathway were replicated and confirmed by qPCR analysis (Figure 9I).

## Conclusions

In the present study, a *S. alba* transcriptome was assembled *de novo* for the first time, to the best of our knowledge. The 47,972 generated unigenes were a mean length of 1185 nt and had an N50 of 1672 nt, indicating a high quality reference transcriptome for genetic studies. The produced 14,727 SSRs will be useful for the genetic analysis of this non-model crop. Although no reference genome was available, 97% of the unigenes were functionally annotated. Expression profiles showed that the root accumulated the largest fraction of specifically and predominantly expressed genes, indicating its involvement in many more specialized functions. The genes predominantly expressed in the root were enriched in pathways related to lignin and cellulose syntheses, plant-pathogen interactions, and pathways potentially responsible for heavy metal chelating and detoxification. The glucosinolate and phytochelatin metabolic pathways, which confer the characteristics and utilities of this plant, were intensively analyzed. The genes encoding aliphatic GSLs were predominantly expressed in the root. The absence of aliphatic GSLs in leaf tissues was most likely due to the lack of *BCAT4* expression and the low expressions of *MAM1* and *CYP79F1*, which efficiently blocked the pathway. Glutathione in the root was extensively converted into phytochelatin, but in the leaf, it was actively converted to its oxidized form. The transcriptome and SSR markers from this study will benefit basic research on and the molecular breeding of *S. alba* and will also be useful for studying the mechanisms of GSLs, phytoremediation and other important traits, as well as the transfer of these beneficial traits to other crops.

## Author contributions

XZ and XL designed the study. XZ, TL, MD, and JS performed the experiments. XZ analyzed the data and drafted the manuscript. All of the authors carefully checked and approved this manuscript.

## Data access

RNAseq data are available at EMBL/NCBI/SRA (accession numbers SRR2961888, SRR2961889, and SRR2961890).

### Conflict of interest statement

The authors declare that the research was conducted in the absence of any commercial or financial relationships that could be construed as a potential conflict of interest.
